# Supplementary material for: Cystatin F (Cst7) drives sex-dependent changes in microglia in an amyloid-driven model of Alzheimer’s disease
Source: eLife. 2023 Dec 12;12:e85279. doi: 10.7554/eLife.85279 (PMC10715728; doi:10.7554/eLife.85279)
Supplement: Figure 2—source data 2. [file elife-85279-fig2-data2.docx]

**A** Male *vs.* Female in *App^NL-G-F^*

| **Up (higher in males)** | | **Down (higher in females)** | |
| --- | --- | --- | --- |
| *Hykk* | *Gpx3* | | ***Ctse*** |
| *Nr1d2* | ***Gpnmb*** | | *Kcnj2* |
| *Ttc7* | *Gpx1* | | *Lrrc27* |
| *Gm14023* | *Rgs16* | | ***Spp1*** |
| *Pan3* | *Phlda3* | | *Gm33858* |
| *Hyou1* | *Mgl2* | | *Kcnk3* |
| *Gm10382* | *Selenow* | | *Tmem51* |
| *Tmem229a* | *N4bp3* | | ***C3*** |
|  |  | | *Cck* |

**B** Male *vs.* Female in *App^NL-G-F^Cst7^-/-^*

| **Up (higher in males)** | | **Down (higher in females) (selected)** | | | |
| --- | --- | --- | --- | --- | --- |
| *Hsp90b1* | *Ccdc71* | *Mir155hg* | *Enpp2* | *Il12b* | *Col4a1* |
| *P4ha1* | *AI506816* | *Vegfa* | *Gpr37l1* | *Gadd45b* | *Rab20* |
| *Plod1* | *Mis12* | *Sox7* | *Armc2* | ***Tnf*** | *Csrnp1* |
| *Entpd1* | *Txndc5* | *Ttr* | *F3* | *Il1rn* | ***Nlrp3*** |
| *P2ry12* | *Stip1* | *Gdf3* | *Acsbg1* | *Gm1673* | *Dcstamp* |
| *Serinc3* | *Hsp90ab1* | *Gdf10* | ***Il1b*** | *Cd69* | *Lilrb4a* |
| *Selplg* | *Creld2* | *Id3* | *Hbegf* | *Lrguk* | *Cdkn1a* |
| *P2ry6* | *Rnf19a* | *Gpat2* | *Phlda1* | *Hes1* | *Tnfaip2* |
| *Pdia6* | *Pomt2* | ***Cxcl1*** | *Tamalin* | ***Nfkbid*** | *Nfkbiz* |
| *Tnfrsf11a* | *Exog* | ***Cxcl2*** | *Lzts3* | *Selenom* | *Insig1* |
| *P2ry13* | *Cog3* | *1200007C1* | *H2-K2* | *Sdc4* | *Pim3* |
| *Rpn1* | *Sec23ip* | *Nr4a1* | *Tmem178* | *Tmem51* | *Gem* |
| *Slc35e1* | *Tprn* | *Rgs11* | *Phlda3* | *Ccrl2* | *Gpx1* |
| *Pros1* | *Hyou1* | *Slc39a2* | *Bcl2a1d* | *Cd83* | *Miip* |
| *Eif2ak1* | *Zscan22* | *Ifrd1* | *Kcnh2* | ***Gpnmb*** | *Marcksl1* |
| *Fkbp4* | *Zfp516* | *H2-Q5* | *Maff* | *Tnfaip3* | *Selenoh* |
| *Tm9sf2* | *Katnb1* | *Dusp2* | *Snhg15* | ***Il1a*** | *Rel* |
| *Dusp6* | *Pigg* | *Mgl2* | *Gpx3* | *Gm13889* | *Vcam1* |
| *Pdia4* |  | *Tnfsf9* | *Kdm6b* | *Osm* | ***Spp1*** |
| *Txndc11* |  | *Selenow* | *Siglec1* | *Tob1* | *Id2* |
|  |  | *Dusp5* | *Cxcl10* | *Rasgef1b* | *Nfkbia* |
